# Case in Which a Child Swallowed an Iron Nail, about an Inch Long

**Published:** 1825-06

**Authors:** W. Thomas

**Affiliations:** Member of the Royal College of Surgeons, &c.


ART. VII.—*Case in which a Child swallowed an Iron Nail, about an Inch long.* By W. THOMAS, Esq. Member of the Royal College of Surgeons, &c.

SATURDAY, 3d April, 1824.—I was summoned, in great haste, to M. Naptun, an infant eighteen months old, who had just before swallowed an iron nail, about an inch long. On my arrival, I found the child in strong convulsions, which occurred immediately after the nail had slipped into the throat. On examining the fauces, no portion of it could be discovered; and being large, and having a rough head, I hesitated a moment to give an emetic, but ordered a warm bath, which soon relieved the convulsions. Spontaneous vomiting ensuing, I aided it by the administration of warm water; yet the irritating cause remained in the stomach, but could not be felt externally, even when that organ was quite empty. The infant being in some measure relieved, absolute rest, in a recumbent posture, was insisted on; keeping the bowels mildly and regularly open.

On the fourth day, the stools became black, and evidently ferruginous; with a retention of urine. This being relieved, all went on well, till the seventh day, (without any portion of iron passing,) when a similar train of symptoms arose,—viz. vomiting and convulsions; which, however, gave way, in an hour or two, to the same measures as at first adopted. Gradual improvement now took place till the fourteenth day, when the infant suffered for a few hours, as in the first instance; and the symptoms again recurred on the twentieth. During the intervals her health was very good, free from pain; she sucked well, and slept naturally. From this period the child improved rapidly, although no appearance (other than the dark coloured stools on the fourth day,) of the nail's passing from the bowels could be discovered, although the motions were regularly watched for many weeks.

Query.—Is it not likely these fits of suffering, at such regular intervals, were brought on, after the first day, by the nail's attempts to pass the pylorus? The case, I think, tends to show the power the gastric juice has of dissolving substances in the stomach; it having evidently dissolved the iron nail, with comparatively little derangement of the natural functions.

*Pembroke Dock; April 21st, 1825.*

